# PCA via joint graph Laplacian and sparse constraint: Identification of differentially expressed genes and sample clustering on gene expression data

**DOI:** 10.1186/s12859-019-3229-z

**Published:** 2019-12-30

**Authors:** Chun-Mei Feng, Yong Xu, Mi-Xiao Hou, Ling-Yun Dai, Jun-Liang Shang

**Affiliations:** 1 Bio-Computing Research Center, Harbin Institute of Technology, Shenzhen, 518055 Guangdong People’s Republic of China; 2 School of Information Science and Engineering, Qufu Normal University, Rizhao, 276826 People’s Republic of China; 3 Key Laboratory of Network Oriented Intelligent Computation, Shenzhen, 518055 People’s Republic of China

**Keywords:** Differentially expressed genes, Gene expression data, Graph Laplacian, Principal component analysis, Sparse constraint

## Abstract

**Background:**

In recent years, identification of differentially expressed genes and sample clustering have become hot topics in bioinformatics. Principal Component Analysis (PCA) is a widely used method in gene expression data. However, it has two limitations: first, the geometric structure hidden in data, e.g., pair-wise distance between data points, have not been explored. This information can facilitate sample clustering; second, the Principal Components (PCs) determined by PCA are dense, leading to hard interpretation. However, only a few of genes are related to the cancer. It is of great significance for the early diagnosis and treatment of cancer to identify a handful of the differentially expressed genes and find new cancer biomarkers.

**Results:**

In this study, a new method gLSPCA is proposed to integrate both graph Laplacian and sparse constraint into PCA. gLSPCA on the one hand improves the clustering accuracy by exploring the internal geometric structure of the data, on the other hand identifies differentially expressed genes by imposing a sparsity constraint on the PCs.

**Conclusions:**

Experiments of gLSPCA and its comparison with existing methods, including Z-SPCA, GPower, PathSPCA, SPCArt, gLPCA, are performed on real datasets of both pancreatic cancer (PAAD) and head & neck squamous carcinoma (HNSC). The results demonstrate that gLSPCA is effective in identifying differentially expressed genes and sample clustering. In addition, the applications of gLSPCA on these datasets provide several new clues for the exploration of causative factors of PAAD and HNSC.

## Background

In the field of bioinformatics, research on the difference of expressed genes between cells of different status helps us understand the functions of genes, and what is more, isolate disease related genes. This direction of research is known as identification of differentially expressed genes [1]. It lays a foundation for further research on the relationship between cancer and genes in the molecular level and will improve the efficiency of cancer diagnosis. Sample clustering of gene expression data is another application in bioinformatics [2, 3]. It will facilitate the searching of new cancer subtype, and consequently helps the targeted therapy of tumor.

Matrix decomposition is one of the major techniques to deal with gene expression data [4–8]. At present, many researchers are interested in data modeling, and try to select the differentially expressed genes from a large number of gene expression data [9–12]. It lays a foundation for further research on the relationship between cancer and genes in the molecular level and improves the efficiency of cancer diagnosis. Among them, Principal Component Analysis (PCA) is a basic tool that has been widely used [6, 7]. The traditional linear PCA method considers the global Euclidean structure of the original data, and when the data points are in a manifold structure, the global Euclidean structure cannot exactly describe the real distance between data points.

In recent years, manifold learning has made a lot of progress in theory, algorithm and application [13–16]. The main idea of manifold learning is to establish a nonlinear mathematical model by means of differential calculus and other mathematical tools. The inherent nonlinear geometric structure hidden in the high dimensional data can be revealed by manifold learning. Thus, we can consider introducing the manifold learning in the linear PCA method. Motivated by manifold learning theory, Jiang et al. proposed graph Laplacian PCA (gLPCA) [17]. This method joins a graph Laplacian to the data representation of original data **X**. The derived low dimensional data can be learned with the cluster information encoded in graph structure **W**.

Despite its advantage, the PCA joint with graph Laplacian suffers from the fact that the PCs are typically dense [18, 19]. In bioinformatics, the gene expression data involved in PCs have much irrelevant or redundant information. For the study of the pathogenesis of a disease, only a small number of genes are significant. This information plays an important role in early diagnosis of cancer. Hence, the interpretation of the PCs will be facilitated if the derived PCs are sparse, involving a few of nonzero elements. Actually, many sparse PCA methods have been developed. For example, rotation and thresholding are first derived on running and facial spots data to find the sparse PCs [20, 21]. Z-SPCA is designed based on iterative elastic net regression [22]. Good results on biological and regular multivariate data have been achieved by this method. D’Aspremont et al. designed two methods, called DSPCA [23] and PathSPCA [24]. DSPCA finds sparse PCs via semi-definite program (SDP) while PathSPCA directly identifies the nonzero elements one by one. Shen and Huang designed a method called sPCA-rSVD which solves the problem based on low-rank matrix factorization [25]. Sigg and Buhmann considered expectation-maximization (EM) to solve a sparse probabilistic generative model, we call this method as EMSPCA [26]. Journée et al. designed a series of algorithms based on L0 and L1-norm to extract single unit or block unit PCs (GPowerL0, GPowerL1, GPowerL0,m, GPowerL1,m,) on random data and gene expression data to compute the sparse PCs [27]. Lai et al. rewrote the traditional PCA into multilinear regression and sparse regression forms (MSPCA) to deal with tensor data [28]. Motivated by rotation and truncation of PCA basis, Hu et al. proposed an efficient method called SPCArt [29]. Zhao et al. divided the sparse PCA problem into several sub-problems and gave a series of closed-form solutions to compute it. This method is named as block coordinate descent sparse PCA (BCD-SPCA) [30].

Recently, Nie et al. demonstrated that L2,1-norm applying on a matrix can induce sparsity in row [18, 31]. L2,1-norm is defined as ${\left\Vert \mathbf{X}\right\Vert}_{2,1}={\sum}_{\mathrm{i}=1}^{\mathrm{n}}\sqrt{\sum_{j=1}^m{\mathbf{x}}_{ij}^2}={\sum}_{i=1}^n{\left\Vert {\mathbf{x}}^i\right\Vert}_2$, where **x**^*i*^ is the i-th row of **X**. Actually L2,1-norm first computes L2-norm of the row vector **x**^*i*^ and then calculates L1-norm of the resulting L2-norms *b*(**X**) = (‖**x**^1^‖_2_, ‖**x**^2^‖_2_, ...‖**x**^*m*^‖_2_). Zero rows in **X** can be achieved through the effect of L2,1-norm. Thus, considering manifold learning has little effect on identification of differentially expressed genes, the introduction of L2,1-norm to PCA is feasible and effective. Furthermore, as we will show, when the problem is solved iteratively, L2,1-norm can be formulated into a trace form, consequently we can optimize it compatibly with the graph Laplacian.

In this paper, we consider introducing sparsity constraint and graph Laplacian to PCA. A novel method called PCA via joint graph Laplacian and sparse constraint (gLSPCA) is proposed. It not only encodes with the internal geometric structure for clustering purpose, but also imposes sparse constraint on traditional PCA to improve interpretability. As a result, on one hand our method can be applied for sample clustering; on the other hand, it can identify a few of differentially expressed genes. The contributions of this paper can be enumerated as follows:

(i) We proposed a novel method called gLSPCA which simultaneously learns the internal geometric structure and improves the interpretability of PCs. gLSPCA on the one hand can identify differentially expressed genes, on the other hand can be applied for sample clustering.
(ii) The optimization and convergence analysis of gLSPCA are provided.
(iii) The proposed gLSPCA is effective in identifying differentially expressed genes and sample clusters, as demonstrated by experimental results on PAAD and HNSC datasets. gLSPCA provides a tool that is helpful for the study of the pathogenesis of cancer, and the clustering application on samples provides a basis for early diagnosis of cancer.

In what follows, the proposed method and the algorithm for this method are introduced in Methodology section. The properties and convergence analysis of this method are included. Extensive experiments for differentially expressed genes identification and sample clustering are conducted in the section of Results and Discussion, where related sparse PCA methods are compared with our method. The paper is concluded in the section of Conclusions.

## Methodology

### Mathematical definition

Above all, we define some notations which will be frequently used in following sections. (1) The input data matrix is denoted by **X** = (*x*_1_, ..., *x*_*n*_) ∈ *ℝ*^*m* × *n*^, where *n* is the number of samples and *m* is the number of variables, i.e., genes in the gene expression data. (2) The new subspace of projected data points is denoted by **H** = *ℝ*^*n* × *k*^ and the principal direction is denoted by **U** = (**u**_1_, ..., **u**_*k*_) ∈ *ℝ*^*m* × *k*^. (3) The Frobenius norm is denoted as ‖**X**‖_*F*_. (4) The L_2,1_-norm is denoted as ${\left\Vert \mathbf{X}\right\Vert}_{2,1}={\sum}_{\mathrm{i}=1}^{\mathrm{n}}\sqrt{\sum_{j=1}^m{\mathbf{x}}_{ij}^2}={\sum}_{i=1}^n{\left\Vert {\mathbf{x}}^i\right\Vert}_2$. (4) The trace of matrix **Z** is denoted as Tr(**Z**).

### The classical PCA and Graph-Laplacian PCA

In this subsection, we briefly review the classical PCA and gLPCA. PCA finds the new subspace of projected data points **H** and principal direction **U** by solving the following optimization problem [7]:

1 $$ \underset{\mathbf{U},\mathbf{H}}{\min }{\left\Vert \mathbf{X}-\mathbf{U}{\mathbf{H}}^T\right\Vert}_F^2\kern1em s.t.\kern0.3em {\mathbf{H}}^T\mathbf{H}=\mathbf{I}. $$

In gene expression data, each column *x*_*i*_ is a linearized vector of sample. The basic PCA model cannot recover non-linear structure of data. gLPCA incorporates the geometric manifold information to find the non-linear structure of data [7]. Considering **H** is the embedding matrix, the gLPCA is formulated as follows:

2 $$ \underset{\mathbf{U},\mathbf{H}}{\min }{\left\Vert \mathbf{X}-\mathbf{U}{\mathbf{H}}^T\right\Vert}_F^2+\alpha \mathrm{Tr}\left({\mathbf{H}}^T\mathbf{LH}\right)\kern1.2em s.t.\kern0.4em {\mathbf{H}}^T\mathbf{H}=\mathbf{I}, $$

where **L** = **D** − **W** is the graph Laplacian matrix. **D** =  *diag* (*d*_1_, ..., *d*_*n*_) is a diagonal matrix whose elements are column or row sums of **W** (**W** is a symmetric nonnegative weight matrix). It can be expressed as *d*_*i*_ = ∑_*j*_**W**_*ij*_. The definition of **W**_*ij*_ is listed as follows:

3 $$ {\mathbf{W}}_{ij}=\Big\{{\displaystyle \begin{array}{l}1\kern1.7em if\kern0.3em {\mathbf{x}}_i\in {\mathbf{N}}_k\left({\mathbf{x}}_j\right)\kern0.6em or\kern0.5em {\mathbf{x}}_j\in {\mathbf{N}}_k\left({\mathbf{x}}_i\right),\\ {}0\kern1.5em otherwise,\end{array}}\kern1em $$

where **N**_*k*_(**x**_*i*_) is the *k* nearest neighbours of **x**_*i*_ [24]. The authors also presented a robust version to improve the robustness of this method. Since our paper focuses on the sparsity of the gLPCA method, we will not elaborate this robust version further.

### The proposed method: PCA via joint graph Laplacian and sparse regularization (gLSPCA)

Recently, sparse representation has been widely applied in the field of bioinformatics. It decomposes a set of high-dimensional data into a series of linear combinations of low dimensional codes, and hopes the combination coefficients to be zero as much as possible. The PCA suffers from the fact that the PCs are typically dense. The interpretation of the PCs might be facilitated if the idea of sparse constraint has been utilized. We consider introducing L_2,1_-norm constraint on the PCs **H** to improve the interpretability of PCA based method. Since the L_2,1_-norm can induce sparsity in rows, the PCs can be sparser and more easily explained [25]. Then, the quality of the decomposition is improved. The proposed method (gLSPCA) solves the following minimization problem:

4 $$ \underset{\mathbf{U},\mathbf{H}}{\min }{\left\Vert \mathbf{X}-\mathbf{U}{\mathbf{H}}^T\right\Vert}_F^2+\alpha \mathrm{Tr}\left({\mathbf{H}}^T\mathbf{LH}\right)+\gamma {\left\Vert \mathbf{H}\right\Vert}_{2,1}\kern1.1em s.t.\kern0.4em {\mathbf{H}}^T\mathbf{H}=\mathbf{I}, $$

where *α* and *γ* are scalar parameters to balance the weights of graph Laplacian and sparse constraint respectively.

### Optimization

It is hard to obtain a closed solution from Eq. (4). Thus, we solve the problem via iterative optimization. The solution of **U** is obtained by calculating partial derivatives at first. Then, the solution of **H** can be obtained by performing eigen-decomposition, after these two variables **U** and **H** are integrated into one variable **H** to substitute the original objective function. Obtaining convergence after a number of iterations, we finally get the PCs with internal geometry and sparsity which were ignored in previous studies. Firstly, following an optimization technique of L2,1-norm [25, 26], the optimization of original problem can be approximated by the following problem:

5 $$ \underset{\mathbf{U},\mathbf{H}}{\min }{\left\Vert \mathbf{X}-\mathbf{U}{\mathbf{H}}^T\right\Vert}_F^2+\alpha \mathrm{Tr}\left({\mathbf{H}}^T\mathbf{LH}\right)+\gamma \mathrm{Tr}\left({\mathbf{H}}^T\mathbf{DH}\kern0.1em \right)\kern0.9000001em s.t.\kern0.4em {\mathbf{H}}^T\mathbf{H}=\mathbf{I}, $$

where **D** is a diagonal matrix with elements:

6 $$ {\mathbf{D}}_{ii}=\frac{1}{2{\left\Vert {\mathbf{h}}_i\right\Vert}_2}. $$

Then, to get the solution of **U**, we fix **H** and the derivative of *ℒ*(**U**, **H**, **D**) respect to **U** is

7 $$ \frac{\partial \mathcal{L}\left(\mathbf{U},\mathbf{H},\mathbf{D}\right)}{\partial \mathbf{U}}=-2\mathbf{XH}+2\mathbf{U}, $$

By setting the derivative of **U** to zero, we have

8 $$ \mathbf{U}=\mathbf{XH}. $$

Substituting the solutions of **U** into Eq. (5), we have

9 $$ {\displaystyle \begin{array}{l}\mathrm{Tr}\left(\mathbf{X}-\mathbf{XH}{\mathbf{H}}^T\right){\left(\mathbf{X}-\mathbf{XH}{\mathbf{H}}^T\right)}^T+\alpha \mathrm{Tr}\left({\mathbf{H}}^T\mathbf{LH}\right)+\gamma \mathrm{Tr}\left({\mathbf{H}}^T\mathbf{DH}\right)\\ {}=-\mathrm{Tr}\left({\mathbf{H}}^T{\mathbf{X}}^T\mathbf{X}\mathbf{H}\right)+{\left\Vert \mathbf{X}\right\Vert}_F^2+\alpha \mathrm{Tr}\left({\mathbf{H}}^T\mathbf{LH}\right)+\gamma \mathrm{Tr}\left({\mathbf{H}}^T\mathbf{DH}\right)\\ {}=\mathrm{Tr}\left({\mathbf{H}}^T\left(-{\mathbf{X}}^T\mathbf{X}+\alpha \mathbf{L}+\gamma \mathbf{D}\right)\mathbf{H}\right)+{\left\Vert \mathbf{X}\right\Vert}_F^2.\end{array}} $$

Therefore, Eq. (8) is equivalent to the following problem:

10 $$ \ell \left(\mathbf{H}\right)=\underset{{\mathbf{H}}^T\mathbf{H}=\mathbf{I}}{\min}\mathrm{Tr}\left({\mathbf{H}}^T\mathbf{AH}\kern0.1em \right), $$

where **A** =  − **X**^*T*^**X** + *α***L** + *γ***D**. Thus, the optimal **H** is the eigenvectors corresponding to the first *k* smallest eigenvalues of the matrix **A**.

In the following, for convenience of parameter setting, we transform **A** to another equivalent form. We use *η*_*k*_ to denote the largest eigenvalue of matrix **X**^*T*^**X** − *γ***D**. For Laplacian matrix **L**, we use *η*_*s*_ to represent the largest eigenvalue of **L**. We then set

11 $$ \alpha =\frac{\beta }{1-\beta}\frac{\eta_k}{\eta_s}, $$

so that the tuning of *α* becomes the tuning of *β*. Thus, (4) can be rewritten as follows:

12 $$ \underset{\mathbf{H}}{\min}\mathrm{Tr}\ {\mathbf{H}}^T\left[\left(1-\beta \right)\left(\mathbf{I}-\frac{{\mathbf{X}}^T\mathbf{X}+\gamma \mathbf{D}}{\eta_k}\right)+\beta \frac{\mathbf{L}}{\eta_s}\kern0.1em \right]\kern0.1em \mathbf{H}\kern1.9em s.t.\kern0.6em {\mathbf{H}}^T\mathbf{H}=\mathbf{I}. $$

In this way, the solution of **H** can be obtained by computing the first *k* smallest eigenvalues of matrix **A**_1_:

12 $$ {\mathbf{A}}_1=\left(1-\beta \right)\left(\mathbf{I}-\frac{{\mathbf{X}}^T\mathbf{X}+\gamma \mathbf{D}}{\eta_k}\right)+\beta \frac{\mathbf{L}}{\eta_s}. $$

The range of *β* is 0 ≤ *β* ≤ 1. In particular, when *β* = 0 and *γ* = 0, gLSPCA degrades to classical PCA. When *β* = 1 and *γ* = 0, it equals to Laplacian Embedding (LE). We summarize the algorithm of the proposed gLSPCA approach in Algorithm 1.

**Table:** 

**Input**: Data matrix **X** = (*x*_1_, ..., *x*_*n*_) ∈ *R*^*m* × *n*^, parameters *γ* and *β*.	
**Output**: Matrix **U** and **H**.	
**1**: Initialize **D** = **I**_*n* × *n*_;	
**2**: **repeat**	
Construct weight matrix **W**; Compute the diagonal matrix **D**, graph Laplacian **L**; Compute **H** by the eigenvectors corresponding to the first *k* smallest eigenvalues of matrix **A**_1_; Compute the optimal **U** according to Eq. (8); Compute diagonal matrix **D** according to Eq. (6);	
**Until converges**	

### Convergence analysis

We would like to show the objective value does not increase in each iteration of the proposed gLSPCA algorithm. Firstly, a simple lemma is provided [32].

#### Lemma 1.

For any non-zero vectors **a,b** ∈ *ℝ*^*m*^:

14 $$ {\left\Vert \mathbf{a}\right\Vert}_2-\frac{{\left\Vert \mathbf{a}\right\Vert}_2^2}{2{\left\Vert \mathbf{b}\right\Vert}_2}\le {\left\Vert \mathbf{b}\right\Vert}_2-\frac{{\left\Vert \mathbf{b}\right\Vert}_2^2}{2{\left\Vert \mathbf{b}\right\Vert}_2}. $$

The convergence analysis of gLSPCA is summarized as Theorem 1.

#### Theorem 1:

The optimization procedure of the proposed gLSPCA algorithm will monotonically decrease the objective function in each iteration.

#### Proof.

Following the algorithm of gLSPCA, when we fix **D**^*t*^ in the *t*-th iteration and optimize **U**^*t* + 1^, **H**^*t* + 1^, we have:

15 $$ {\displaystyle \begin{array}{l}{\left\Vert \mathbf{X}-{\mathbf{U}}^{t+1}{\left({\mathbf{H}}^{t+1}\right)}^T\right\Vert}_F^2+\alpha \mathrm{Tr}\left({\left({\mathbf{H}}^{t+1}\right)}^T\mathbf{L}{\mathbf{H}}^{t+1}\right)+\gamma \mathrm{Tr}\left({\mathbf{H}}^{t+1}{\mathbf{D}}^t{\mathbf{H}}^{t+1}\kern0.1em \right)\\ {}\le {\left\Vert \mathbf{X}-{\mathbf{U}}^t{\left({\mathbf{H}}^t\right)}^T\right\Vert}_F^2+\alpha \mathrm{Tr}\left({\left({\mathbf{H}}^t\right)}^T\mathbf{L}{\mathbf{H}}^t\right)+\gamma \mathrm{Tr}\left({\mathbf{H}}^t{\mathbf{D}}^t{\mathbf{H}}^t\kern0.1em \right).\end{array}} $$

Since ${\left\Vert \mathbf{H}\right\Vert}_{2,1}={\sum}_{i=1}^n{\left\Vert {\mathbf{h}}_i\right\Vert}_2$, this inequality indicates

16 $$ {\displaystyle \begin{array}{l}{\left\Vert \mathbf{X}-{\mathbf{U}}^{t+1}{\left({\mathbf{H}}^{t+1}\right)}^T\right\Vert}_F^2+\alpha \mathrm{Tr}\left({\left({\mathbf{H}}^{t+1}\right)}^T\mathbf{L}{\mathbf{H}}^{t+1}\right)+\gamma \sum \limits_{i=1}^n\left(\frac{{\left\Vert {\mathbf{h}}_i^{t+1}\right\Vert}_2^2}{2{\left\Vert {\mathbf{h}}_i^t\right\Vert}_2}-{\left\Vert {\mathbf{h}}_i^{t+1}\right\Vert}_2\right)\\ {}\le {\left\Vert \mathbf{X}-{\mathbf{U}}^t{\left({\mathbf{H}}^t\right)}^T\right\Vert}_F^2+\alpha \mathrm{Tr}\left({\left({\mathbf{H}}^t\right)}^T\mathbf{L}{\mathbf{H}}^t\right)+\gamma \sum \limits_{i=1}^n\left(\frac{{\left\Vert {\mathbf{h}}_i^t\right\Vert}_2^2}{2{\left\Vert {\mathbf{h}}_i^t\right\Vert}_2}-{\left\Vert {\mathbf{h}}_i^{t+1}\right\Vert}_2\right).\end{array}} $$

According to Lemma 1, we know that

17 $$ \frac{{\left\Vert {\mathbf{h}}_i^{t+1}\right\Vert}_2^2}{2{\left\Vert {\mathbf{h}}_i^t\right\Vert}_2}-{\left\Vert {\mathbf{h}}_i^{t+1}\right\Vert}_2\ge \frac{{\left\Vert {\mathbf{h}}_i^t\right\Vert}_2^2}{2{\left\Vert {\mathbf{h}}_i^t\right\Vert}_2}-{\left\Vert {\mathbf{h}}_i^t\right\Vert}_2. $$

Thus, we have the following result.

18 $$ {\displaystyle \begin{array}{l}{\left\Vert \mathbf{X}-{\mathbf{U}}^{t+1}{\left({\mathbf{H}}^{t+1}\right)}^T\right\Vert}_F^2+\alpha \mathrm{Tr}\left({\left({\mathbf{H}}^{t+1}\right)}^T\mathbf{L}{\mathbf{H}}^{t+1}\right)+\gamma {\left\Vert {\mathbf{H}}^{t+1}\right\Vert}_{2,1}\\ {}\le {\left\Vert \mathbf{X}-{\mathbf{U}}^t{\left({\mathbf{H}}^t\right)}^T\right\Vert}_F^2+\alpha \mathrm{Tr}\left({\left({\mathbf{H}}^t\right)}^T\mathbf{L}{\mathbf{H}}^t\right)+\gamma {\left\Vert {\mathbf{H}}^t\right\Vert}_{2,1}.\end{array}} $$

This inequality proves that the objective function of (4) will monotonically decrease in each iteration.

## Results and discussion

The primary goal of our method is to improve the sparsity of gLPCA because the PCs of this method are dense. We evaluate the performance of the proposed method with the other five related methods, including four sparse PCA methods: Z-SPCA [22], GPower [27], PathSPCA [24], SPCArt [29], and a graph Laplacian PCA method: gLPCA [17]. There are two deflation algorithms and two block algorithms for GPower. In practice, the results of the four algorithms are not different significantly. We choose one of these algorithms as comparison method in our experiments. The experiments are mainly divided into two aspects:

(i) Identifying differentially expressed genes. The joint effect of sparse constraint and graph Laplacian in our method can be evaluated by the identification of differentially expressed genes. Firstly, the new oncogenes can be found in these discovered differentially expressed genes. Then, the function and interacting proteins network analysis of these new oncogenes are given. Finally, pathway analysis explains the combined biological processes of the identified differentially expressed genes.
(ii) Tumour sample clustering. Since the sparse PCs are encoded with the internal geometric structure for clustering purpose, tumour sample clustering can be used to evaluate how well it works. Clustering the data according to the similarity of each data point provides a basis for accurate subtype of cancer.

### Experimental settings

We set *r* = 2 to be the number of reduced dimensions. The similarity matrix is constructed by k-nearest neighbour graph with Gaussian kernel, where we set *k* = 5 and the *σ* of Gaussian kernel to be 1. We set *λ* to infinity as the parameter value of Z-SPCA method, thus soft thresholding can be conducted to compute the sparse PCs for the gene expression data with high dimension and small sample. For GPower and PathSPCA method, we use the default parameter values suggested by the authors. For SPCArt method, we set ${\lambda}^{\ast }=1/\sqrt{m}$ to guarantee the sparsity and avoid truncating to zero vectors. For our method, the best parameters are selected in the range of *β* = (0.1, ..., 0.9) and *γ* = (10^−30^, ..., 10^30^). We report the best results with the optimal parameters for all compared methods.

### Datasets

The details of the two datasets used in our experiments are described in Table 1. The dataset of pancreatic cancer (PAAD) and head and neck squamous carcinoma (HNSC) are downloaded from The Cancer Genome Atlas (TCGA). This database is an open comprehensive multi-dimensional map of the key genomic changes in 33 types of cancer dataset. These two datasets have thousands of genes but only a small number of samples. Much irrelevant or redundant information is contained in such gene expression data. The following experiments on identification of differentially expressed genes and tumour sample clustering are particularly important in the cancer study.

**Table 1:** Summary of the two datasets

Data sets	Number of		class distribution	
	Samples	Genes	Normal	disease
PAAD	180	20,502	4	176
HNSC	418	20,502	20	398

### Identifying differentially expressed genes

In bioinformatics, the PCs involve a large number of genes. In cancer study, only a small number of genes are significant for early diagnosis of cancer and accurate subtype of cancer. These genes can be defined as differentially expressed genes. We can analyze the identified differentially expressed genes to evaluate the effectiveness of the sparsity constraint in our method.

Firstly, we compute the scores for all genes in descending order. Then, the index set of differentially expressed genes is formed by the corresponding indices. To be fair, all methods extract the largest 100 values. The extracted genes with high scores in data representation can be deemed as differentially expressed genes. We match the selected differentially expressed genes to the pathogenic genes of PAAD and HNSC published on GeneCards. The public available website of GeneCards is http://www.genecards.org/, which is an open, integrative database that provides comprehensive, useful information on all predicted and annotated human genes [33].

Matching results of each method on PAAD and HNSC datasets are listed in an additional file (see Additional file 1). Additional file 1 shows the differentially expressed genes identified by all compared methods, as well as the relative scores of each gene associated with the disease. The unique genes of each method are also marked in bold in this file. These unique genes are the differentially expressed genes that one method can identify while the other methods cannot.

**Fig. 1:**
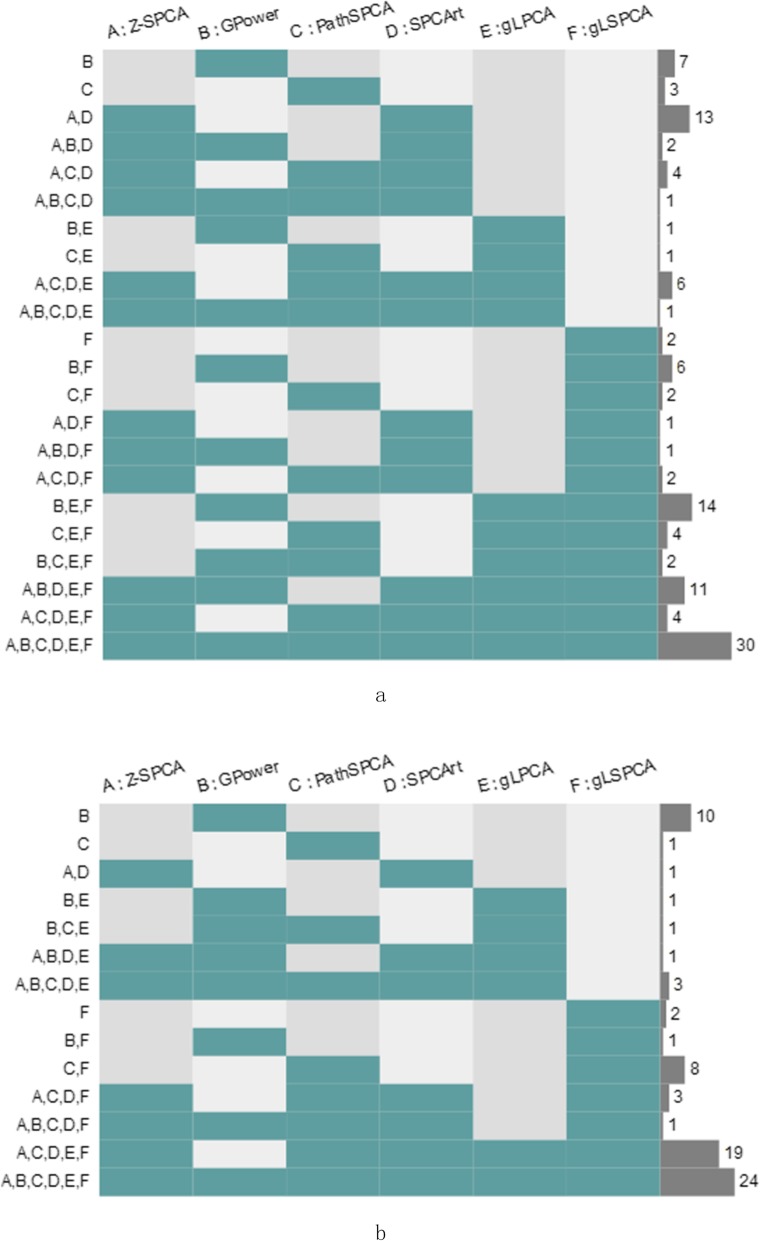
Overlap among the differentially expressed genes identified by the compared methods

To visualize the overlap among the differentially expressed genes identified by the methods, we send the results to OmicsBean to generate a Venn diagram. OmicsBean is a multi-group data analysis system, and its public address is http://www.omicsbean.com:88/. The overlap result of the differentially expressed genes identified by the methods is visualized by Venn diagram in Fig. 1. In this figure, (a) is the overlap result on PAAD dataset and (b) is the overlap result on HNSC dataset. The left coordinate represents the different permutations and combinations of various methods. The right coordinate represents the number of unique genes that are excavated by one method (only one method on the left coordinate) or the same genes by several methods (multiple methods on the left coordinate).

From Fig. 1, it can be concluded that the number of unique gene mined by GPower on the two datasets is the most. But it also loses a large number of important genes, which can be seen from the results of the Additional file 1. These missing genes lead to a poor overall effect in identifying differentially expressed genes. There are also a few unique genes mined by PathSPCA. But the scores of these genes are not high enough, they are not important pathogenic genes. Thus, there is no further research of these genes. gLSPCA finds two unique genes in each dataset, and these genes are highly related to disease. These genes can be defined as the new oncogenes identified by our method. It is necessary to carry out further studies on these genes and their detailed analysis is discussed in the next subsection.

Here, we first detect the efficiency of the identified differentially expressed genes. The identification accuracy (IA) and the total relevance scores (TRS) of these genes are listed in Table 2. The best results are in bold. The IA is defined as follows:

19 $$ IA=\frac{\Sigma_{i=1}^n\psi \left({g}_s,{g}_n\right)}{n}\times 100\%, $$

where *ψ*(*x*, *y*) = 1 if *x* = *y* and *ψ*(*x*, *y*) = 0 otherwise. *g*_*s*_ is the selected genes from our method and *g*_*n*_ represents the pathogenic genes of disease from GeneCards. Larger IA indicates the identification performance of differentially expressed genes is better. TRS denotes the total relevance scores of all identified differentially expressed genes, which is computed by GeneCards.

**Table 2:** Results on identification accuracy (IA) and total relevance score (TRS) of six methods on PAAD and HNSC dataset

Methods	PAAD		HNSC	
	IA	TRS	IA	TRS
Z-SPCA	77.00	901.67	53.00	540.91
GPower	77.00	922.27	43.00	378.70
PathSPCA	61.00	682.56	**60.00**	579.06
SPCArt	77.00	901.67	53.00	540.91
gLPCA	75.00	878.39	50.00	513.94
gLSPCA	**80.00**	**927.70**	**60.00**	**591.31**

From this table, it can be concluded that gLSPCA has higher IA and TRS results than the other methods over the two datasets. The results of Z-SPCA, SPCArt and gLPCA methods are relatively stable, while those of GPower and PathSPCA methods are unstable. The IA result of PathSPCA on HNSC equals to that of gLSPCA, but the result on PAAD dataset is the worst. Since the differentially expressed genes identified by our method have higher correlation with disease, the TRS of PathSPCA is lower than gLSPCA. For GPower method, the IA and TRS performances have much difference on the two datasets. It shows that the adaptability of GPower to different datasets is not satisfactory. From the above discussion, we can conclude that the proposed method gLSPCA performs better than the other methods on identifying differentially expressed genes.

### Function and interacting proteins network analysis

To detect the correlation between identified oncogenes with disease, we summarize the functions of these genes in Tables 3 and 4. Table 3 lists the function of differentially expressed genes on PAAD dataset identified by gLSPCA but not the other methods. The relevance score of PPY on PAAD indicates that it is an important virulence gene of PAAD. Published article has proved that PPY responses to a mixed meal in PAAD [34]. Thus, the medical study of PAAD is based on the biological changes of PPY. CD24 as a potential oncogene interferes the RNA treatment of PAAD cancer cells has been studied [35]. And its relevance score with PAAD is 8.27. Table 4 lists the functions and relevance scores of the differentially expressed genes on HNSC dataset identified by gLSPCA but not the other methods. The high relevance scores reflect the close relationship between HSPA1A, COL6A1 and HNSC. But there are few biological researches on this issue, which provides a great space to study it.

**Table 3:** The function of differentially expressed genes on PAAD dataset identified by gLSPCA but not the other methods

Gene name	Function	Relevance score
PPY	This gene encodes a member of the neuropeptide Y (NPY) family of peptides.	21.13
CD24	This gene encodes a sialoglycoprotein that is expressed on mature granulocytes and B cells and modulates growth and differentiation signals to these cells.	8.27

**Table 4:** The function of differentially expressed genes on HNSC dataset identified by gLSPCA but not the other methods

Gene name	Function	Relevance score
HSPA1A	This intronless gene encodes a 70 kDa heat shock protein which is a member of the heat shock protein 70 family.	7.67
COL6A1	The collagens are a superfamily of proteins that play a role in maintaining the integrity of various tissues.	4.05

**Fig. 2:**
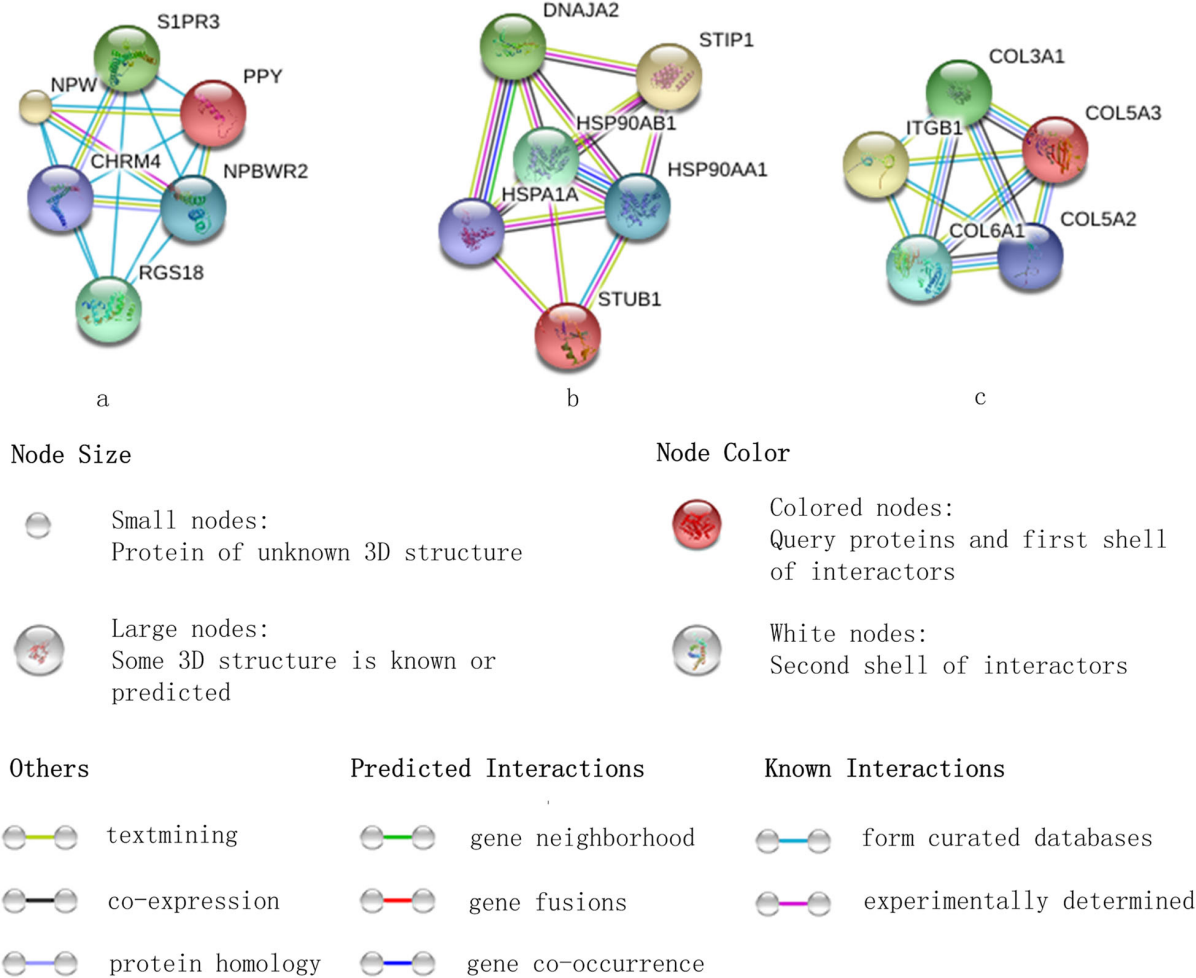
The interacting proteins network of the identified differentially expressed genes Graphical presentation of the interacting proteins network of the differentially expressed genes identified by gLSPCA but not the other compared methods.

Since these genes belong to protein coding genes, it is useful to send them to GeneCards for finding the interacting proteins network. We find three interacting proteins networks. The results can be found in Fig. 2, where (a) is the interacting proteins network of PPY, (b) is HSPA1A and (c) is COL6A1. Each graph shows the most significant five interacting genes. In this figure, each network node represents proteins result by a single and protein-coding gene locus. The edges in this figure are the protein-protein associations. These associative proteins jointly promote a shared function, which does not necessarily mean they are binding each other in physics. The specific known interactions are explained in this figure. The graph of protein networks are helpful to carry out a deeper biological study of these differentially expressed genes.

### Pathway analysis

The combined biological processes of the identified differentially expressed genes can be explained by pathways. Pathways help us understand the advanced functions of organisms and biological systems at the molecular level. We send these genes to KEGG: http://www.kegg.jp/. The pathways of highest overlap on these two datasets are presented in additional file [36]. The pathway of focal adhesion plays an essential role in biological processes. This pathway is discovered from the identified differentially expressed genes of gLSPCA on PAAD dataset. The published article concludes that the modification of focal adhesion and integration might be a novel therapeutic approach for the treatment of pancreatic cancer [37]. The pathway of ECM-receptor interaction is identified by the result of gLSPCA on HNSC dataset. The extracellular matrix (ECM) is made up of various structural and functional macromolecules, and it also plays a vital role in tissue and organ morphogenesis, as well as the holding of cell and tissue structure. Shim et al. hypothesize the over expression of cortical protein leads to the degradation of ECM in HNSC [38]. Researches show the biological system in these pathways is an important part for the study of PAAD and HNSC.

### Tumour sample clustering results

Sample clustering based on gene expression data is helpful for the detection of tumour subtypes. Since the graph Laplacian is introduced to the proposed method, the geometric structure of data is explored. However, the encoded internal geometric structure is for clustering purpose. It is useful to evaluate whether the explored geometric structure benefits sample clustering. And the discovery new subtype of tumour is helpful for the targeted therapy of tumour. In this experiment, filtering out redundant information by the proposed method, the corresponding results are obtained by K-means clustering. Following the related clustering work, we adopt clustering accuracy (ACC) as evaluation criteria in our experiments [39]. The criteria of ACC can be calculated by

20 $$ ACC=\frac{\Sigma_{i=1}^n\delta \left({p}_i, map\left({q}_i\right)\right)}{n}\times 100\%, $$

where *q*_*i*_ is the clustering label obtained by the algorithm and *p*_*i*_ is the truth label. *δ*(*p*_*i*_, *map*(*q*_*i*_)) is given by

21 $$ \delta \left(x,y\right)=\Big\{{\displaystyle \begin{array}{l}1,\kern1.1em x=y,\\ {}0,\kern1.1em otherwise,\end{array}} $$

where *map*(*q*_*i*_) is the best mapping function. Table 5 summarizes the ACC results of all compared methods, in which “All-Ge” denotes all gene clusters without any dimension reduction processing. The best results are highlighted in bold. From the results, the observations can be summarized as follows:

(i) Since the graph Laplacian is introduced to gLSPCA for clustering purpose, the gLSPCA method performs better than the other methods.
(ii) All-Ge has the lowest ACC result on PAAD dataset, and has intermediate result on HNSC. On PAAD dataset, the clustering results might be interfered by too much irrelevant and redundant information if dimensionality reduction is not employed. However, seldom irrelevant and redundant information contained in HNSC dataset, as well as much information loss in some sparse PCA methods, might lead to the intermediate result of All-Ge on this dataset.

**Table 5:** ACC performance of all methods

Datasets	All-Ge	Z-SPCA	GPower	PathSPCA	SPCArt	gLPCA	gLSPCA
PAAD	83.09	95.00	95.00	95.00	96.35	95.00	**97.22**
HNSC	78.23	75.84	77.51	72.73	75.84	79.43	**92.88**

Notes: “All-Ge” denotes all features cluster without any dimension reduction processing

## Conclusions

In this paper, we have proposed a new PCA method called gLSPCA by joint graph Laplacian and sparse constraint. The most distinguished characteristics of the new method are that gLSPCA not only considers the internal geometric structure in the data representation, but also adds sparse constraint to PCA. Specifically, we obtain PCs to represent the data meanwhile transform the PCs to approximate the cluster membership indicators in K-means method. The algorithm as well as the convergence analysis of this method has also been developed. The effectiveness of our method has been demonstrated on differentially expressed genes identification and tumour sample clustering comparing with currently available sparse and graph based PCA methods. Finally, we have evaluated the identified differentially expressed genes in the way of co-expression (pathways) and interacting proteins network.

## Supplementary information

**Additional file 1.** The pathways of highest overlap on PAAD and HNSC datasets, the pathway of focal adhesion and ECM-receptor interaction. Matching results of each method on PAAD and HNSC datasets.

## Abbreviations

ACC: clustering accuracy
All-Ge: denotes all genes cluster without any dimension reduction processing
BCD_SPCA: block coordinate descent sparse PCA
DSPCA and PathSPCA: two sparse PCA method designed by D’Aspremont
EMSPCA: expectation-maximization sparse PCA
gLSPCA: PCA via joint graph Laplacian and Sparse regularization
GPower: a serious of sparse PCA method based on L_0_- and L_1_-norm with single unit or block units (GPower_L0,_ GPower_L1,_ GPower_L0,m,_ GPower_L1,m,_)
HNSC: head and neck squamous carcinoma
IA: differentially expressed genes identification performance
MSPCA: Multilinear regression and Sparse regression PCA
PAAD: pancreatic cancer
PCA: Principal component analysis
PCs: Principal Components
SDP: semidefinite program
sPCA-rSVD: low-rank matrix factorization sparse PCA
SPCArt: sparse PCA Motivated based on rotation matrix and sparse basis
TCGA: The Cancer Genome Atlas
TRS: total relevance scores
Z-SPCA: a sparse PCA method designed by Zou et al.
