# Supplementary material for: PCA via joint graph Laplacian and sparse constraint: Identification of differentially expressed genes and sample clustering on gene expression data
Source: BMC Bioinformatics. 2019 Dec 30;20(Suppl 22):716. doi: 10.1186/s12859-019-3229-z (PMC6936054; doi:10.1186/s12859-019-3229-z)
Supplement: Supplementary file 1 — Additional file 1. The pathways of highest overlap on PAAD and HNSC datasets, the pathway of focal adhesion and ECM-receptor interaction. Matching results of each method on PAAD and HNSC datasets. [file 12859_2019_3229_MOESM1_ESM.docx]

**Additional files：**

Notes: These two tables list the differentially expressed genes identified by all compared methods on two datasets, and the relative scores of each gene associated with the disease. Among them, the red is a unique gene for one method.


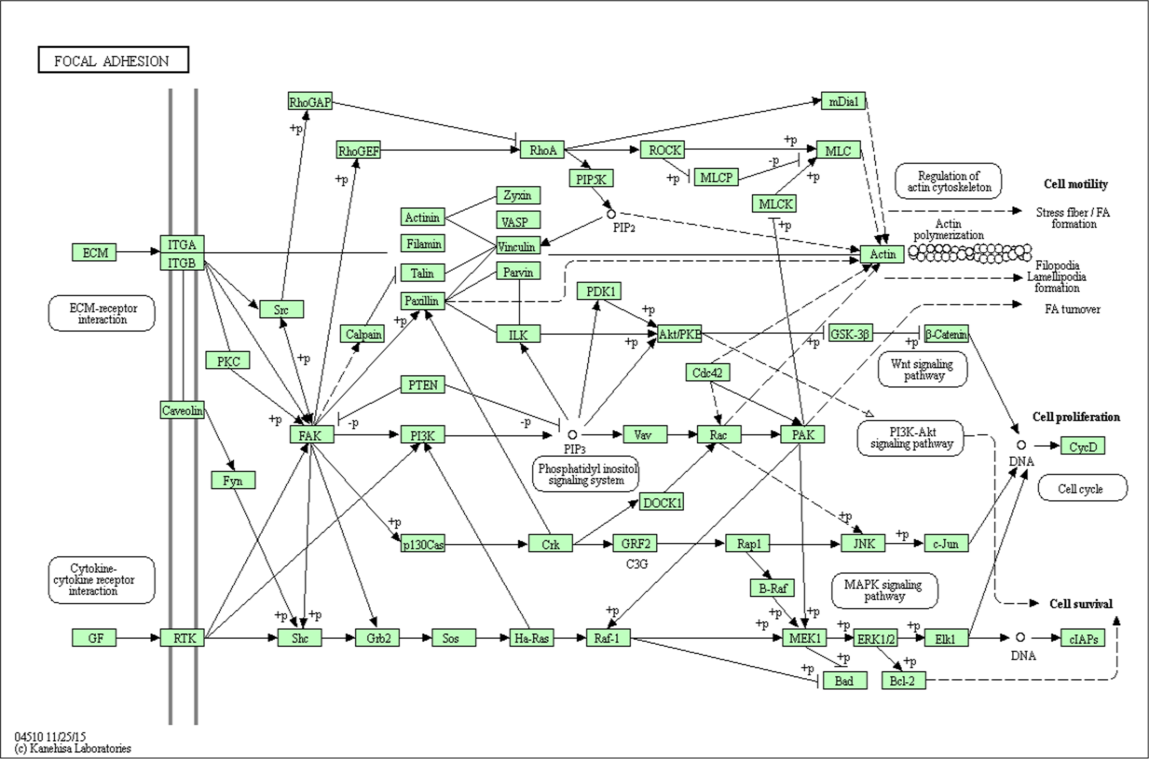


The pathway of focal adhesion. Graphical presentation of the essential role of focal adhesion plays in biological processes.


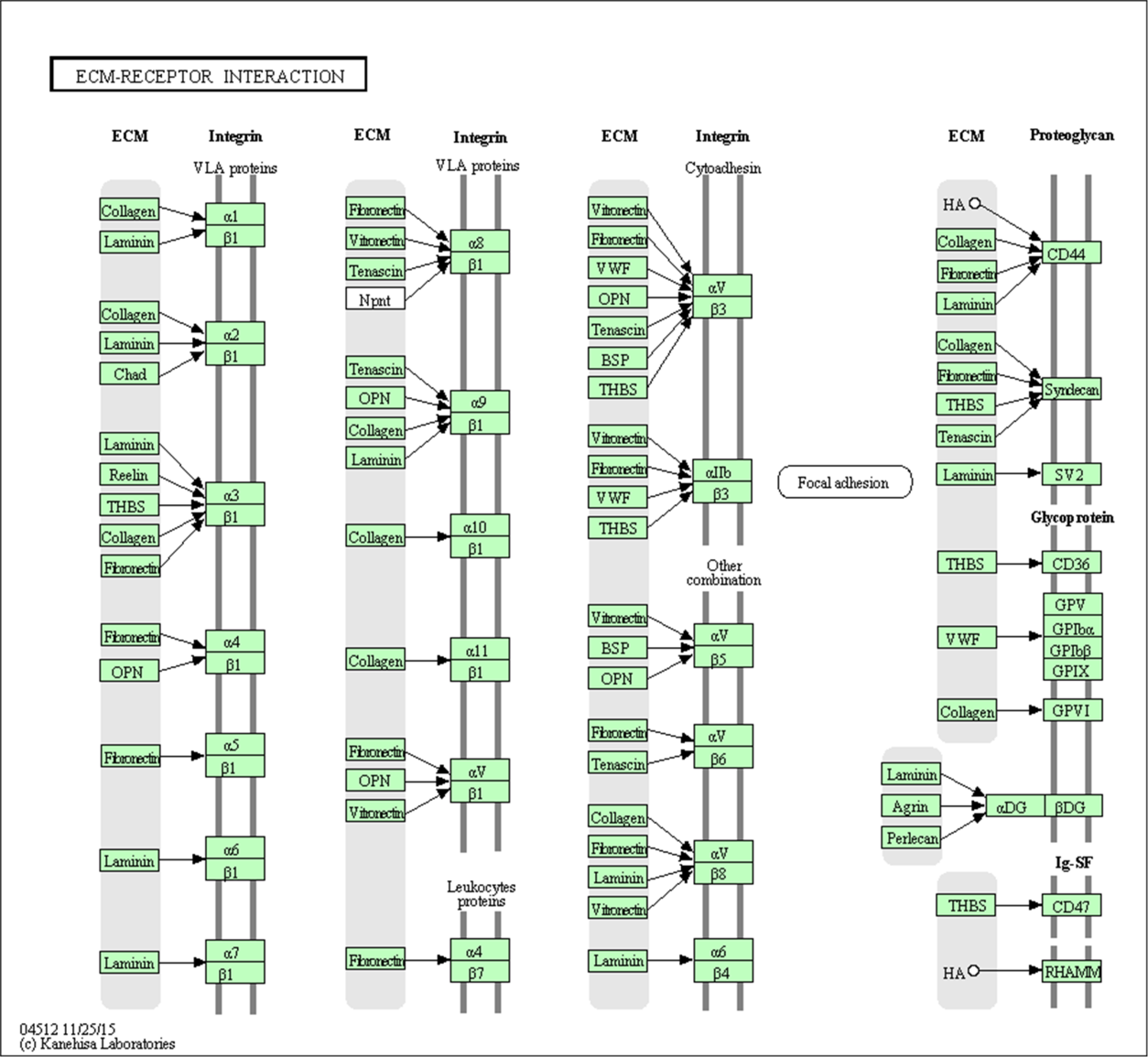


The pathway of ECM-receptor interaction. The extracellular matrix (ECM) is made up of various structural and functional macromolecules, and it also plays a vital role in tissue and organ morphogenesis, as well as the holding of cell and tissue structure and function.

result on HNSC dataset

| Z-SPCA | Relevance score | GPower | Relevance score | PathSPCA | Relevance score | SPCArt | Relevance score | GLPCA | Relevance score | GLSPCA | Relevance score |
| --- | --- | --- | --- | --- | --- | --- | --- | --- | --- | --- | --- |
| CD44 | 37.28 | KRT19 | 27.05 | CD44 | 37.28 | CD44 | 37.28 | CD44 | 37.28 | CD44 | 37.28 |
| GSTP1 | 32.16 | KRT5 | 21.85 | GSTP1 | 32.16 | GSTP1 | 32.16 | GSTP1 | 32.16 | GSTP1 | 32.16 |
| SLC2A1 | 29.08 | KRT14 | 21.33 | SLC2A1 | 29.08 | SLC2A1 | 29.08 | SLC2A1 | 29.08 | SLC2A1 | 29.08 |
| KRT5 | 21.85 | FN1 | 17.81 | KRT5 | 21.85 | KRT5 | 21.85 | KRT5 | 21.85 | KRT19 | 27.05 |
| KRT14 | 21.33 | GJB2 | 17.25 | KRT14 | 21.33 | KRT14 | 21.33 | KRT14 | 21.33 | KRT5 | 21.85 |
| FN1 | 17.81 | KRT10 | 16.96 | FN1 | 17.81 | FN1 | 17.81 | FN1 | 17.81 | KRT14 | 21.33 |
| CTSD | 17.42 | KRT13 | 16.62 | CTSD | 17.42 | CTSD | 17.42 | HLA-B | 17.38 | FN1 | 17.81 |
| HLA-B | 17.38 | LY6D | 15.88 | HLA-B | 17.38 | HLA-B | 17.38 | GJB2 | 17.25 | CTSD | 17.42 |
| GJB2 | 17.25 | CXCL14 | 14.32 | GJB2 | 17.25 | GJB2 | 17.25 | KRT10 | 16.96 | HLA-B | 17.38 |
| KRT10 | 16.96 | HSPB1 | 13.02 | KRT13 | 16.62 | KRT10 | 16.96 | KRT13 | 16.62 | GJB2 | 17.25 |
| KRT13 | 16.62 | S100A2 | 11.43 | HSP90AA1 | 16.62 | KRT13 | 16.62 | HLA-A | 16.55 | KRT13 | 16.62 |
| HSP90AA1 | 16.62 | CSTA | 10.85 | HLA-A | 16.55 | HSP90AA1 | 16.62 | SDC1 | 14.89 | HSP90AA1 | 16.62 |
| HLA-A | 16.55 | IVL | 9.09 | SDC1 | 14.89 | HLA-A | 16.55 | CTSB | 13.61 | HLA-A | 16.55 |
| SDC1 | 14.89 | S100A9 | 8.87 | CTSB | 13.61 | SDC1 | 14.89 | HSPB1 | 13.02 | SDC1 | 14.89 |
| CTSB | 13.61 | SFN | 8.82 | HSPB1 | 13.02 | CTSB | 13.61 | SPARC | 12.76 | CTSB | 13.61 |
| HSPB1 | 13.02 | ANXA1 | 8.72 | SPARC | 12.76 | HSPB1 | 13.02 | S100A2 | 11.43 | HSPB1 | 13.02 |
| SPARC | 12.76 | KRT4 | 8.22 | H19 | 11.51 | SPARC | 12.76 | LAMC2 | 11.06 | SPARC | 12.76 |
| S100A2 | 11.43 | PKP1 | 7.71 | S100A2 | 11.43 | S100A2 | 11.43 | CSTA | 10.85 | H19 | 11.51 |
| LAMC2 | 11.06 | KRT1 | 7.57 | LAMC2 | 11.06 | LAMC2 | 11.06 | ANXA2 | 9.14 | S100A2 | 11.43 |
| ANXA2 | 9.14 | SLPI | 7.27 | ITGA6 | 10.52 | ANXA2 | 9.14 | S100A9 | 8.87 | LAMC2 | 11.06 |
| S100A9 | 8.87 | PKM | 7.22 | ANXA2 | 9.14 | S100A9 | 8.87 | SFN | 8.82 | ITGA6 | 10.52 |
| SFN | 8.82 | KRT16 | 6.98 | S100A9 | 8.87 | SFN | 8.82 | ANXA1 | 8.72 | ANXA2 | 9.14 |
| ANXA1 | 8.72 | DSG3 | 6.88 | SFN | 8.82 | ANXA1 | 8.72 | KRT4 | 8.22 | S100A9 | 8.87 |
| KRT4 | 8.22 | S100A7 | 6.84 | ANXA1 | 8.72 | KRT4 | 8.22 | PLEC | 8.18 | SFN | 8.82 |
| PLEC | 8.18 | KLK10 | 5.91 | KRT4 | 8.22 | PLEC | 8.18 | PKP1 | 7.71 | ANXA1 | 8.72 |
| PKP1 | 7.71 | TGM3 | 5.84 | PLEC | 8.18 | PKP1 | 7.71 | KRT1 | 7.57 | PLEC | 8.18 |
| KRT1 | 7.57 | LGALS7 | 5.56 | TUBB | 8.03 | KRT1 | 7.57 | HLA-C | 7.46 | TUBB | 8.03 |
| HLA-C | 7.46 | COL1A1 | 5.56 | PKP1 | 7.71 | HLA-C | 7.46 | PKM | 7.22 | PKP1 | 7.71 |
| PKM | 7.22 | YWHAZ | 5.36 | KRT1 | 7.57 | PKM | 7.22 | KRT16 | 6.98 | HSPA1A | 7.67 |
| KRT16 | 6.98 | KRT6A | 5.16 | HLA-C | 7.46 | KRT16 | 6.98 | DSG3 | 6.88 | HLA-C | 7.46 |
| DSG3 | 6.88 | DSG1 | 5.1 | PKM | 7.22 | DSG3 | 6.88 | S100A7 | 6.84 | PKM | 7.22 |
| ENO1 | 6.61 | DSP | 4.94 | KRT16 | 6.98 | ENO1 | 6.61 | ENO1 | 6.61 | KRT16 | 6.98 |
| GNAS | 6.23 | PI3 | 4.93 | DSG3 | 6.88 | GNAS | 6.23 | LDHA | 6.14 | DSG3 | 6.88 |
| LDHA | 6.14 | CSTB | 4.58 | S100A7 | 6.84 | LDHA | 6.14 | CFL1 | 5.56 | ENO1 | 6.61 |
| CFL1 | 5.56 | KRT15 | 4.3 | ENO1 | 6.61 | CFL1 | 5.56 | COL1A1 | 5.56 | LDHA | 6.14 |
| COL1A1 | 5.56 | CRNN | 3.9 | LDHA | 6.14 | COL1A1 | 5.56 | YWHAZ | 5.36 | APP | 5.71 |
| YWHAZ | 5.36 | ACTG1 | 3.75 | APP | 5.71 | YWHAZ | 5.36 | KRT6A | 5.16 | TUBA1B | 5.58 |
| KRT6A | 5.16 | KRT17 | 3.25 | TUBA1B | 5.58 | KRT6A | 5.16 | DSP | 4.94 | CFL1 | 5.56 |
| DSP | 4.94 | TGM1 | 3.12 | CFL1 | 5.56 | DSP | 4.94 | PI3 | 4.93 | COL1A1 | 5.56 |
| PI3 | 4.93 | FTL | 2.56 | COL1A1 | 5.56 | PI3 | 4.93 | CD74 | 4.79 | YWHAZ | 5.36 |
| CD74 | 4.79 | JUP | 2.44 | YWHAZ | 5.36 | CD74 | 4.79 | CSTB | 4.58 | KRT6A | 5.16 |
| CSTB | 4.58 | KRT6B | 2.16 | KRT6A | 5.16 | CSTB | 4.58 | ACTG1 | 3.75 | DSP | 4.94 |
| ACTG1 | 3.75 | COL17A1 | 1.72 | DSP | 4.94 | ACTG1 | 3.75 | ALDOA | 3.42 | PI3 | 4.93 |
| ALDOA | 3.42 | total socres | 378.7 | PI3 | 4.93 | ALDOA | 3.42 | P4HB | 3.38 | CD74 | 4.79 |
| P4HB | 3.38 |  |  | CD74 | 4.79 | P4HB | 3.38 | KRT17 | 3.25 | LAMB3 | 4.51 |
| KRT17 | 3.25 |  |  | CSTB | 4.58 | KRT17 | 3.25 | B2M | 2.91 | KRT15 | 4.3 |
| B2M | 2.91 |  |  | LAMB3 | 4.51 | B2M | 2.91 | MYH9 | 2.78 | COL6A1 | 4.05 |
| MYH9 | 2.78 |  |  | ACTG1 | 3.75 | MYH9 | 2.78 | JUP | 2.44 | ACTG1 | 3.75 |
| FTL | 2.56 |  |  | ITGB4 | 3.61 | FTL | 2.56 | KRT6B | 2.16 | ALDOA | 3.42 |
| JUP | 2.44 |  |  | ALDOA | 3.42 | JUP | 2.44 | COL17A1 | 1.72 | P4HB | 3.38 |
| KRT6B | 2.16 |  |  | P4HB | 3.38 | KRT6B | 2.16 | total socres | 513.94 | TAGLN2 | 3.3 |
| PSAP | 1.83 |  |  | TAGLN2 | 3.3 | PSAP | 1.83 |  |  | KRT17 | 3.25 |
| COL17A1 | 1.72 |  |  | KRT17 | 3.25 | COL17A1 | 1.72 |  |  | B2M | 2.91 |
| total socres | 540.91 |  |  | B2M | 2.91 | total socres | 540.91 |  |  | MYH9 | 2.78 |
|  |  |  |  | MYH9 | 2.78 |  |  |  |  | FTL | 2.56 |
|  |  |  |  | FTL | 2.56 |  |  |  |  | JUP | 2.44 |
|  |  |  |  | JUP | 2.44 |  |  |  |  | KRT6B | 2.16 |
|  |  |  |  | KRT6B | 2.16 |  |  |  |  | PSAP | 1.83 |
|  |  |  |  | PSAP | 1.83 |  |  |  |  | TNC | 1.73 |
|  |  |  |  | TNC | 1.73 |  |  |  |  | COL17A1 | 1.72 |
|  |  |  |  | COL17A1 | 1.72 |  |  |  |  | total socres | 591.31 |
|  |  |  |  | total socres | 579.06 |  |  |  |  |  |  |

result on PAAD dataset

| Z-SPCA | Relevance score | GPower | Relevance score | PathSPCA | Relevance score | SPCArt | Relevance score | GLPCA | Relevance score | GLSPCA | Relevance score |
| --- | --- | --- | --- | --- | --- | --- | --- | --- | --- | --- | --- |
| CD44 | 37.28 | KRT19 | 27.05 | CD44 | 37.28 | CD44 | 37.28 | CD44 | 37.28 | CD44 | 37.28 |
| GSTP1 | 32.16 | KRT5 | 21.85 | GSTP1 | 32.16 | GSTP1 | 32.16 | GSTP1 | 32.16 | GSTP1 | 32.16 |
| SLC2A1 | 29.08 | KRT14 | 21.33 | SLC2A1 | 29.08 | SLC2A1 | 29.08 | SLC2A1 | 29.08 | SLC2A1 | 29.08 |
| KRT5 | 21.85 | FN1 | 17.81 | KRT5 | 21.85 | KRT5 | 21.85 | KRT5 | 21.85 | KRT19 | 27.05 |
| KRT14 | 21.33 | GJB2 | 17.25 | KRT14 | 21.33 | KRT14 | 21.33 | KRT14 | 21.33 | KRT5 | 21.85 |
| FN1 | 17.81 | KRT10 | 16.96 | FN1 | 17.81 | FN1 | 17.81 | FN1 | 17.81 | KRT14 | 21.33 |
| CTSD | 17.42 | KRT13 | 16.62 | CTSD | 17.42 | CTSD | 17.42 | HLA-B | 17.38 | FN1 | 17.81 |
| HLA-B | 17.38 | LY6D | 15.88 | HLA-B | 17.38 | HLA-B | 17.38 | GJB2 | 17.25 | CTSD | 17.42 |
| GJB2 | 17.25 | CXCL14 | 14.32 | GJB2 | 17.25 | GJB2 | 17.25 | KRT10 | 16.96 | HLA-B | 17.38 |
| KRT10 | 16.96 | HSPB1 | 13.02 | KRT13 | 16.62 | KRT10 | 16.96 | KRT13 | 16.62 | GJB2 | 17.25 |
| KRT13 | 16.62 | S100A2 | 11.43 | HSP90AA1 | 16.62 | KRT13 | 16.62 | HLA-A | 16.55 | KRT13 | 16.62 |
| HSP90AA1 | 16.62 | CSTA | 10.85 | HLA-A | 16.55 | HSP90AA1 | 16.62 | SDC1 | 14.89 | HSP90AA1 | 16.62 |
| HLA-A | 16.55 | IVL | 9.09 | SDC1 | 14.89 | HLA-A | 16.55 | CTSB | 13.61 | HLA-A | 16.55 |
| SDC1 | 14.89 | S100A9 | 8.87 | CTSB | 13.61 | SDC1 | 14.89 | HSPB1 | 13.02 | SDC1 | 14.89 |
| CTSB | 13.61 | SFN | 8.82 | HSPB1 | 13.02 | CTSB | 13.61 | SPARC | 12.76 | CTSB | 13.61 |
| HSPB1 | 13.02 | ANXA1 | 8.72 | SPARC | 12.76 | HSPB1 | 13.02 | S100A2 | 11.43 | HSPB1 | 13.02 |
| SPARC | 12.76 | KRT4 | 8.22 | H19 | 11.51 | SPARC | 12.76 | LAMC2 | 11.06 | SPARC | 12.76 |
| S100A2 | 11.43 | PKP1 | 7.71 | S100A2 | 11.43 | S100A2 | 11.43 | CSTA | 10.85 | H19 | 11.51 |
| LAMC2 | 11.06 | KRT1 | 7.57 | LAMC2 | 11.06 | LAMC2 | 11.06 | ANXA2 | 9.14 | S100A2 | 11.43 |
| ANXA2 | 9.14 | SLPI | 7.27 | ITGA6 | 10.52 | ANXA2 | 9.14 | S100A9 | 8.87 | LAMC2 | 11.06 |
| S100A9 | 8.87 | PKM | 7.22 | ANXA2 | 9.14 | S100A9 | 8.87 | SFN | 8.82 | ITGA6 | 10.52 |
| SFN | 8.82 | KRT16 | 6.98 | S100A9 | 8.87 | SFN | 8.82 | ANXA1 | 8.72 | ANXA2 | 9.14 |
| ANXA1 | 8.72 | DSG3 | 6.88 | SFN | 8.82 | ANXA1 | 8.72 | KRT4 | 8.22 | S100A9 | 8.87 |
| KRT4 | 8.22 | S100A7 | 6.84 | ANXA1 | 8.72 | KRT4 | 8.22 | PLEC | 8.18 | SFN | 8.82 |
| PLEC | 8.18 | KLK10 | 5.91 | KRT4 | 8.22 | PLEC | 8.18 | PKP1 | 7.71 | ANXA1 | 8.72 |
| PKP1 | 7.71 | TGM3 | 5.84 | PLEC | 8.18 | PKP1 | 7.71 | KRT1 | 7.57 | PLEC | 8.18 |
| KRT1 | 7.57 | LGALS7 | 5.56 | TUBB | 8.03 | KRT1 | 7.57 | HLA-C | 7.46 | TUBB | 8.03 |
| HLA-C | 7.46 | COL1A1 | 5.56 | PKP1 | 7.71 | HLA-C | 7.46 | PKM | 7.22 | PKP1 | 7.71 |
| PKM | 7.22 | YWHAZ | 5.36 | KRT1 | 7.57 | PKM | 7.22 | KRT16 | 6.98 | HSPA1A | 7.67 |
| KRT16 | 6.98 | KRT6A | 5.16 | HLA-C | 7.46 | KRT16 | 6.98 | DSG3 | 6.88 | HLA-C | 7.46 |
| DSG3 | 6.88 | DSG1 | 5.1 | PKM | 7.22 | DSG3 | 6.88 | S100A7 | 6.84 | PKM | 7.22 |
| ENO1 | 6.61 | DSP | 4.94 | KRT16 | 6.98 | ENO1 | 6.61 | ENO1 | 6.61 | KRT16 | 6.98 |
| GNAS | 6.23 | PI3 | 4.93 | DSG3 | 6.88 | GNAS | 6.23 | LDHA | 6.14 | DSG3 | 6.88 |
| LDHA | 6.14 | CSTB | 4.58 | S100A7 | 6.84 | LDHA | 6.14 | CFL1 | 5.56 | ENO1 | 6.61 |
| CFL1 | 5.56 | KRT15 | 4.3 | ENO1 | 6.61 | CFL1 | 5.56 | COL1A1 | 5.56 | LDHA | 6.14 |
| COL1A1 | 5.56 | CRNN | 3.9 | LDHA | 6.14 | COL1A1 | 5.56 | YWHAZ | 5.36 | APP | 5.71 |
| YWHAZ | 5.36 | ACTG1 | 3.75 | APP | 5.71 | YWHAZ | 5.36 | KRT6A | 5.16 | TUBA1B | 5.58 |
| KRT6A | 5.16 | KRT17 | 3.25 | TUBA1B | 5.58 | KRT6A | 5.16 | DSP | 4.94 | CFL1 | 5.56 |
| DSP | 4.94 | TGM1 | 3.12 | CFL1 | 5.56 | DSP | 4.94 | PI3 | 4.93 | COL1A1 | 5.56 |
| PI3 | 4.93 | FTL | 2.56 | COL1A1 | 5.56 | PI3 | 4.93 | CD74 | 4.79 | YWHAZ | 5.36 |
| CD74 | 4.79 | JUP | 2.44 | YWHAZ | 5.36 | CD74 | 4.79 | CSTB | 4.58 | KRT6A | 5.16 |
| CSTB | 4.58 | KRT6B | 2.16 | KRT6A | 5.16 | CSTB | 4.58 | ACTG1 | 3.75 | DSP | 4.94 |
| ACTG1 | 3.75 | COL17A1 | 1.72 | DSP | 4.94 | ACTG1 | 3.75 | ALDOA | 3.42 | PI3 | 4.93 |
| ALDOA | 3.42 | total socres | 378.7 | PI3 | 4.93 | ALDOA | 3.42 | P4HB | 3.38 | CD74 | 4.79 |
| P4HB | 3.38 |  |  | CD74 | 4.79 | P4HB | 3.38 | KRT17 | 3.25 | LAMB3 | 4.51 |
| KRT17 | 3.25 |  |  | CSTB | 4.58 | KRT17 | 3.25 | B2M | 2.91 | KRT15 | 4.3 |
| B2M | 2.91 |  |  | LAMB3 | 4.51 | B2M | 2.91 | MYH9 | 2.78 | COL6A1 | 4.05 |
| MYH9 | 2.78 |  |  | ACTG1 | 3.75 | MYH9 | 2.78 | JUP | 2.44 | ACTG1 | 3.75 |
| FTL | 2.56 |  |  | ITGB4 | 3.61 | FTL | 2.56 | KRT6B | 2.16 | ALDOA | 3.42 |
| JUP | 2.44 |  |  | ALDOA | 3.42 | JUP | 2.44 | COL17A1 | 1.72 | P4HB | 3.38 |
| KRT6B | 2.16 |  |  | P4HB | 3.38 | KRT6B | 2.16 | total socres | 513.94 | TAGLN2 | 3.3 |
| PSAP | 1.83 |  |  | TAGLN2 | 3.3 | PSAP | 1.83 |  |  | KRT17 | 3.25 |
| COL17A1 | 1.72 |  |  | KRT17 | 3.25 | COL17A1 | 1.72 |  |  | B2M | 2.91 |
| total socres | 540.91 |  |  | B2M | 2.91 | total socres | 540.91 |  |  | MYH9 | 2.78 |
|  |  |  |  | MYH9 | 2.78 |  |  |  |  | FTL | 2.56 |
|  |  |  |  | FTL | 2.56 |  |  |  |  | JUP | 2.44 |
|  |  |  |  | JUP | 2.44 |  |  |  |  | KRT6B | 2.16 |
|  |  |  |  | KRT6B | 2.16 |  |  |  |  | PSAP | 1.83 |
|  |  |  |  | PSAP | 1.83 |  |  |  |  | TNC | 1.73 |
|  |  |  |  | TNC | 1.73 |  |  |  |  | COL17A1 | 1.72 |
|  |  |  |  | COL17A1 | 1.72 |  |  |  |  | total socres | 591.31 |
|  |  |  |  | total socres | 579.06 |  |  |  |  |  |  |
